# Perceived Determinants of Excellent Self-Rated Health Among HIV Virally Suppressed Adults in the Eastern Cape Province, South Africa

**DOI:** 10.3390/ijerph23030278

**Published:** 2026-02-24

**Authors:** Zanele Benedict Nomatshila, Laston Gonah, Sibusiso Cyprian Nomatshila, Teke Ruffin Apalata

**Affiliations:** 1 WSU Public Health, Faculty of Medicine and Health Sciences, Walter Sisulu University, Mthatha 5117, South Africa; lgonah@wsu.ac.za (L.G.); snomatshila@wsu.ac.za (S.C.N.); 2 Department of Social Work, Faculty of Law, Humanities and Social Science, Walter Sisulu University, Mthatha 5117, South Africa; 3 Faculty of Health Sciences & Sport, University of Stirling, Stirling FK9 4LA, UK; tapalata@wsu.ac.za; 4 WSU Society and Health Research Institute, Faculty of Medicine and Health Sciences, Walter Sisulu University, Mthatha 5117, South Africa; 5 Department of Laboratory Medicine, Faculty of Medicine and Health Sciences, Walter Sisulu University, Mthatha 5117, South Africa; 6 Center for Clinical Research Advancement Development Leadership and Education, Faculty of Medicine and Health Sciences, Walter Sisulu University, Mthatha 5117, South Africa

**Keywords:** self-rated health, determinants, virally suppressed, people living with HIV, Eastern Cape province

## Abstract

The goal of antiretroviral therapy (ART) is to achieve viral suppression and improve the quality of life in people living with HIV (PLWH). Targeting the determinants of self-rated health in virally suppressed PLWH could significantly contribute towards sustaining the quality of life and health gains from ART. A qualitative study was conducted to investigate the determinants of excellent self-rated health in PLWH who are virally suppressed in the Eastern Cape province. A descriptive cross-sectional study using qualitative approach was conducted among 26 consenting adults living with HIV who have achieved viral load suppression in the rural province of the Eastern Cape using in-depth interviews. Themes were generated from the qualitative data using thematic analysis in NVivo 13^®^. All participants described what they perceived as excellent quality of life as mostly determined by self-system (downward counterfactual thinking and pain discounting), perceived improved health, and adherence to recommended healthy behaviours (ART, diet, physical activity, and non-use of tobacco and alcohol products). Income/financial support availability and good healthcare access emerged as indispensable prerequisites for achieving and maintaining good health or adherence to healthy behaviours. Targeting the determinants of Self-Rated Health (SRH) has the potential to greatly improve physical and mental well-being of PLWH beyond viral suppression. Interventions can be more effective by drawing from evidence generated from context-based research.

## 1. Introduction

The 90-90-90 targets launched by the Joint United Nations programme on HIV/AIDS (UNAIDS) in 2014 sought to direct testing and treatment efforts towards three goals, namely, (1) 90% of people living with HIV (PLWH) being diagnosed and knowing their human immunodeficiency virus (HIV) status, (2) 90% PLWH being on antiretroviral therapy (ART), and (3) 90% of PLWH who are on ART being virally suppressed [1]. Given that concerted efforts (in form of political will and involvement, advocacy, and scientific/technological advancements) have resulted in significant progress towards the achievement of these goals, the focus for the care of PLWH has been recommended to consider the general well-being of PLWH beyond viral suppression [2]. Promoting the well-being of PLWH beyond viral suppression is a holistic approach that aims at ensuring physical and mental well-being, consequently enhancing quality of life improvements in this population.

The goal of ART is to achieve viral suppression and improve the quality of life in PLWH. Beyond viral suppression, virally suppressed PLWH must experience restored health and improved physical, functional, mental, and social well-being, which are important contributing factors for improved quality of life [3]. Hence, beyond the clinical measures of treatment success (e.g., viral suppression), other important aspects that complement a holistic evaluation of an individual’s well-being such as self-rated health (SRH) should be considered.

The SRH is defined as a subjective evaluation of an individual’s health status, or an individual’s perception of their health in general [4]. The SRH has been shown to be associated with objective health outcomes and commented as an effective approach that provides a holistic overview of a person’s current physical and mental well-being [5,6]. The most identified key determinants of SRH include level of education, employment status, viral load and underlying illness, where higher levels of education, having some income/being employed, and being virally suppressed in the absence of persisting/underlying illness(s) were associated with excellent or good SRH [7]. Being a subjective measure, SRH was found to be contextual and to be determined by various factors, which were shown to vary according to individual characteristics (age, level of education, culture/religion, health status, gender, self-system), and socio-economic and environmental factors [6,7,8].

Viral suppression rate for PLWH in South Africa has shown considerable progress towards the global targets, though with some marked disparities [9]. In 2020, 66% of PLWH were virally suppressed globally, with South Africa having achieved 61%, and the Eastern Cape Province of South Africa 58% against a global target of having 73% of all PLWH on ART achieving viral suppression by the year 2020, and 86% by 2025 [10]. Given the indications of increasing viral suppression in PLWH, this progress must be accompanied by corresponding improvements in overall well-being for it to be sustained, particularly among the socially disadvantaged who bear the worst burden of HIV.

The study sought to investigate perceived determinants of excellent SRH in PLWH who are virally suppressed in the Eastern Cape Province, as a critical attempt to generate contextual evidence for informing holistic approaches to promote and sustain well-being in PLWH, beyond viral suppression. This holistic approach will address overall health experiences, integrating the biological, mental, and functional to produce one health. There is a paucity of contextual evidence in the studied population, which has diverse cultural and sociodemographic characteristics.

## 2. Materials and Methods

### 2.1. Study Design

Cross-sectional in-depth qualitative interviews were conducted during the implementation of this study.

### 2.2. Setting

A total of eleven health facilities in the Eastern Cape Province were selected using cluster sampling in order to obtain study participants. The province has a total population of over seven million people, dominated by the Black population (85%) [9]. With over 65% of the provincial population living in non-urban areas, the Eastern Cape province is predominantly rural with high unemployment rates, particularly in the Black population [9]. The interview venue (home or health facility) and time were determined by participant preferences and interviews were performed in the presence of only the interviewer and the interviewee.

### 2.3. Participant Selection

Virally suppressed adult PLWH participated in the study which was conducted between January and June 2023. The study participants were drawn from a population of study participants in a Walter Sisulu University-based study (MRC-RFACC 01-2014) who consented to further studies related to HIV. Purposive sampling was used to select participants and sample size was guided by the principle of data saturation which was achieved at the 26th participant. Both biological sexes had equal chances for participation. Demographic characteristics such as participant age and viral load suppression status were verified for confirmation from patient registers at study enrolment stage.

### 2.4. Data Collection

Data was collected from the 26 participants who met the mentioned eligibility criteria, using a validated in-depth interview guide. Data saturation was determined by non-emergence of new themes from the in-depth interview discussions, following which data collection was stopped when the 26th participant was interviewed. These were conducted by two experienced qualitative study experts with a health background using an indigenous language to ensure participants’ understanding and enhance data richness. The in-depth interviews solicited information on excellent SRH, generating information on how the participants perceived their excellent self-rated health and the corresponding supporting reasons. The in-depth interviews were voice recorded, and this information was complemented by interview notes and session summaries. The interview discussion length ranged between 30 and 45 min. A 95% response rate was achieved on all approached participants with no dropouts experienced.

Sample of questions included the following:

1. In your opinion, what are the most important things that enable a person to achieve and maintain excellent health?
2. What role do you think things like diet, exercise, sleep, or alcohol play?
3. What makes it easy for you to do the things that are good for your health?
4. How does feeling in excellent health allow you to live the life you want to live?

### 2.5. Data Management and Data Analysis

Following the interviews, manual verbatim transcription of recorded data was conducted to generate textual data that was triangulated with data from interview notes and session summaries. Forward (IsiXhosa to English language) and backward (English language to IsiXhosa) translation of the generated textual data was performed to identify any discrepancies and enhance translation quality. The transcribed and translated statements were reviewed by a panel of researchers and participants to prevent misrepresentation and misinterpretation of information, and accuracy was confirmed. Coding and thematic analysis of the yielded textual data was conducted independently by two qualitative research experts using Nvivo version 13^®^. The Lincon and Guba recommendations were used as a reference for ensuring the trustworthiness of the qualitative data [11]. Results from the thematic data analysis were presented according to main emerging themes, and translated verbatim quotes were used to support the themes.

## 3. Results

### 3.1. Participants Characteristics

The study was composed of 26 participants (Table 1) whose ages ranged from 30 to 59 years.

### 3.2. Main Emerging Themes

Data analyses resulted in five main themes being self-system factors, perceived improved health, adherence to recommended healthy behaviour, income/financial support availability and healthcare access as summarised in Table 2. Direct quotes presented are concise, representative, and insightful to maintain balance and reflect participants’ perspectives.

#### 3.2.1. Theme 1: Self-System Factors (Downward Counterfactual Thinking and Pain Discounting)

Participants consistently expressed satisfaction with their current health status compared to the counterfactual possibility/worse possible scenario they could have experienced had they not been on ART and virally suppressed (counterfactual thinking). Those who rated their health as excellent justified their perception that even HIV negative people are prone to some form of illness, and thus they did not consider the minor ailments they experienced from time to time as significant. They reasoned that they could tolerate and expect some form of illness/pain from time to time since they were HIV positive (pain discounting), and since they perceived it as better than the counterfactual possibility of being HIV positive and not being on ART or virally suppressed:

*“My friend, my health is excellent and normal, just like any other [HIV negative] person who is not on [ART] treatment. I am very satisfied because I could have died or been very sick, had I not been on [ART] treatment and virally suppressed. This [HIV] virus got some of my close friends so badly ill that some are already dead and some still very sick as we speak, ……. so being virally suppressed is more than enough for me”*

—Female, 45 years old

*“As for me, I can’t complain. I can safely say my health is good. My health has been better ever since I achieved viral suppression, but being HIV positive, you know that one can fall sick from time to time, but it’s far better compared to what I went through before. Whatever sickness or pain I feel, I think it’s ok……. after all, anyone can fall sick despite the [HIV] status. I go to work, I support my family, and I am happy, so my health is good.”*

—Female, 36 years old

#### 3.2.2. Theme 2: Perceived Improved Health (Experiencing No or Non-Interfering Illness)

Perceived improved health or absence of an underlying condition or persistent pain/illness that interfered with normal day-to-day functions/work emerged as another often-mentioned reason for excellent SRH. Participants reporting good SRH cited having an underlying condition and/or experienced some illness which was reportedly well managed and did not significantly interfere with their daily duties and work:

*“You see, I have been taking my [ART] treatment as prescribed, now I am not sick. I have not experienced any [persistent] sickness for the past 3 months; hence I have not missed going to work. Of course, some minor ailments like flue or headache are common, but that is common to everyone, and I cannot mention that. To answer your question, I see my health as excellent!”*

—Male, 45 years old

*“My health is good, thanks to the [ART] treatment and to this [HIV] negative status [viral suppression]! I also take hypertensive and diabetic medication, so I feel sick from time to time, and sometimes miss going to work for a day or two…. but since I take medications for all those, I have everything well under control and cannot complain.”*

—Female, 55 years old

#### 3.2.3. Theme 3: Adherence to Recommended Healthy Behaviours (ART, Diet, Physical Activity, and Non-Use of Alcohol and Tobacco Products)

Adherence to ART and informed healthy diet and lifestyle behaviours constituted some of the consistently mentioned reasons for an excellent or good SRH. In addition to ART adherence, consumption of a recommended healthy diet or living a healthy lifestyle such as by undertaking some form of physical activity, and non-use of alcohol and tobacco products, were perceived as important complementing factors for an excellent or good SRH:

*“I do not think there is a difference between my health and those who are not on [ART] treatment [people living without HIV]. My health is back to excellent as [I was] before [HIV infection]. It’s because I take the advice I get [from healthcare workers] very seriously: I take my medicine as required… and you see I am now negative [virally suppressed]! I do some exercises, I do not smoke [tobacco cigarettes], I do not drink [alcohol]…… and am [physically] fit as you can see. I am happy too, and not stressed, since I can work for my children and see them grow”*

—Female, 37 years old

*“My health is good because I have seriously adjusted [to diet and lifestyle changes]. I have since quitted smoking [cigarettes] and drinking beer [or alcohol]. I also make it a point that I take my [ART] medication as required, I avoid bad foods [unhealthy diets], and I do some light exercises when I get the time. All this and that I am now negative [virally suppressed] make me happy and no longer worried”*

—Male, 45 years old

#### 3.2.4. Theme 4: Income/Financial Support Availability

All the participants also attributed their excellent or good SRH to economic factors/income. Most of the participants acknowledged that viral suppression had greatly influenced their SRH, enabling them to work and earn some income required for funding their individual health requirements such as healthcare, required medications, diet, and lifestyle behaviours. Unemployed and retired participants reported that a “virally suppressed” status enhanced their functionality to productively undertake piece jobs and earn some income which they need to contribute towards maintaining a health status, complementing the financial support from family and friends:

*“My health is excellent. But I just feel bad for those without money: how can they do what is recommended? We need good [healthy] food, we need money for consultation and for medicine from time to time, and more money to support our families too. As for me, being negative [virally suppressed] has made me fit and able to work and earn some money that I need to fund my needs. If one doesn’t have money though they know what healthy foods they must eat, they will die with their knowledge because they can’t afford the [recommended] lifestyle”*

—Female, 40 years old

*“My health is good. My parent and siblings have been consistent in supporting me with the things I need, such as food, medicine, and all. Now that I feel fit and happy, I guess I must find a job so that I can buy my own things without burdening my relatives”*

—Female, 32 years old

#### 3.2.5. Theme 5: Healthcare Access

The role of the health system in ensuring uninterrupted access to required products and services for enhancing viral suppression and good health was considered a significant determinant of excellent or good SRH.

*“As we speak, my health is excellent, thanks to South Africa’s health services! I get medicines whenever I need it, I get my viral load measurements done, I get advice [from the healthcare workers] on how to live a healthier life. If I could not access all these, I do not think I would say I have an excellent health like I told you”*

—Female, 44 years old

## 4. Discussion

The study investigated the perceived determinants of excellent SRH in virally suppressed PLWH using in-depth interviews, and the most common emerging factors determining excellent SRH among the study participants were (1) self-system factors—counterfactual thinking and pain discounting, (2) perceived improved health—experiencing no or non-interfering illness, (3) adherence to recommended healthy behaviours, (4) availability of income or financial support, and (5) healthcare access.

Self-system factors—counterfactual thinking and pain discounting

The self-system, which constitutes one’s beliefs and self-perceptions or how one interprets personal and environmental circumstances, plays a key role in influencing SRH, which is a subjective measure [6]. The findings show that most participants rated their health based on downward counterfactual possibilities (denoting a worse possible scenario or the acknowledgment that their current health was better than what it could have been had they not been on ART or virally suppressed), resulting in some form of relief or satisfaction [12]. Again, pain discounting was common, where most participants tended to believe that some level of pain or illness was acceptable and better than the worst possible scenario they had not experienced. Scientific and research evidence show that the self-system can either lead to deleterious or beneficial effects to one’s physical and mental well-being, with impaired self-system linked to deleterious effects on physical and mental well-being [8,12]. Being a qualitative study, the study could not establish whether counterfactual thinking or pain discounting varied with participants’ demographic and clinical factors such as age, gender, level of education, level of income, duration on ART or the presence of (an) underlying condition(s). Other studies used quantitative approaches and established that older age and higher educational attainment can improve one’s self-perception and self-efficacy related to their health [13,14].

Perceived improved health—experiencing no or non-interfering illness

In consistence with other study findings, this study found that excellent or good SRH was linked to experiencing no or non-interfering illness and adherence to recommended healthy behaviours [4,7]. Income availability and good healthcare access, just as in principle, emerged as indispensable prerequisites for achieving and maintaining good health or adherence to healthy behaviours [15,16]. Viral suppression is known to promote or to result from improved health, possibly explaining the excellent or good SRH reported from this study.

Adherence to recommended healthy behaviours

Adherence to ART and to recommended diet and lifestyle behaviours have been found to be essential components in promoting and sustaining viral suppression [17,18]. Several study findings have shown that PLWH who do not use alcohol and tobacco products are more likely to achieve HIV viral suppression [17,18,19]. Viral suppression and the reported adherence to healthy diets and lifestyle behaviours such as the non-use of alcohol and tobacco products observed from this study may have promoted good health among study participants, and hence the consistently reported positive SRH.

The determinants of SRH are inextricably linked together in producing the reported SRH outcomes in that no one factor is more important than the other, but the factors interact with each other, resulting in the outcome. For instance, self-system factors such as perceived health status can be influenced by the interaction of one’s personal characteristics (such as age, gender, level of education, level of income, and health status) and past experiences, which can further shape one’s health behaviours, as influenced by the prevailing socio-economic and health system circumstances [8,13,17,18,19]. A pattern can be drawn that predicts SRH: the more negative or bleaker the self-perception on the determinants of SRH is, the more likely that the SRH will be negative. More positive or better self-system factors, better clinical outcomes/experiences, better adherence to recommended healthy behaviours, better economic status, and better healthcare access have consistently predicted better SRH. This trend needs to be quantitatively analysed further and confirmed through studying the factors and performing analyses that were missed by this study. For instance, analysing the role of demographic characteristics and health status (duration on ART, presence of underlying conditions, viral suppression status) on SRH. This will be attempted in subsequent studies linked to this paper.

Availability of income or financial support

It is important to note that income does not act in isolation but serves as a foundational social determinant of health with potential to support or constrain an individual’s ability to access resources, make choices, and live in environments conducive to good SRH [20]. Patients’ financial support plays a significant role in supporting the purchasing of medicines and payments for consultations and laboratory tests and can be a primary reason for disengagement from care [21]. Health systems that provide universal health coverage (UHC) or specific funding for HIV and SRH services has the potential to remove this critical barrier, enabling individuals to initiate and adhere to treatment in the long term without facing financial ruin [22].

Healthcare access

The role of functional health system in ensuring uninterrupted access to the required standard of care treatment is important in ensuring good health of recipients. This can be achieved by training, supporting, and holding the workforce to standards of compassionate, equitable care [23]. This form of continuity is essential for preventing collapse in treatment effects whilst ensuring proper management of drug interactions and coordinating care among healthcare providers [24].

## 5. Conclusions

The findings suggest that positive SRH is not solely an outcome of clinical success like viral suppression but is also shaped by individuals’ psychological framing of their health, their ability to maintain healthy behaviours, and their socio-economic enabling environment. Targeting the determinants of excellent SRH has the potential to greatly improve and sustain the physical and mental well-being of PLWH, beyond viral suppression. Policies need to be structured so that they reflect plans for the sustainability and maintenance of health gains beyond viral load suppression through the integration of psychosocial support into standard care and use of person-centred healthcare access. Individualised behaviour changes and interventions need to be adopted and enacted within frameworks. Interventions can be more effective by drawing from evidence generated from context-based research. Subsequent studies linked to this study can quantitatively assess the association between these demographic and clinical variables and SRH to further explain conundrums reported. They would further investigate the role of demographic (age, gender, educational attainment, marital status) and clinical (duration on ART) factors on SRH, using quantitative approaches and broader inclusivity.

## 6. Strengths and Limitations

The study was conducted in a homogeneous population. Convenient data collection points, as determined by participants, were used. However, through its qualitative nature, findings cannot be generalised as they subjectively reflect the insights of a specific population. Use of only the virally suppressed population has also been identified as a limitation. Achieving saturation at the 20th participant has also been identified as a limiting factor as a larger sample size could enhance the transferability of our findings.

## Figures and Tables

**Table 1:** Demographic characteristics of study participants (*n* = 26).

Variable	Frequency (*n*)	Percentage (%)
**Sex**		
Male	2	8
Female	24	92
**Employment**		
Formal employment	5	19
Self-employment	4	15
Unemployed	16	62
Pensioner	1	4

**Table 2:** Saturation grid of emerging themes on the determinants of excellent self-rated health (SRH) among virally suppressed PLWH (*n* = 26).

Themes	In-Depth Interview Number																									
	1	2	3	4	5	6	7	8	9	10	11	12	13	14	15	16	17	18	19	20	21	22	23	24	25	26
1. Self-system factors (downward counterfactual thinking and pain discounting)	*	*	*	*	*	*	*	*	*	*	*	*	*	*	*	*	*	*	*	*	*	*	*	*	*	*
2. Perceived improved health (experiencing no or non-interfering illness)	*	*	*	*	*	*		*	*	*	*	*	*	*	*	*	*	*	*		*	*	*	*	*	*
3. Adherence to recommended healthy behaviours (ART, diet, physical activity, and non-use of alcohol or tobacco products)	*	*	*	*	*		*	*	*	*	*	*	*	*	*	*	*	*		*	*	*	*		*	*
4. Income/financial support availability	*	*	*	*	*	*	*	*	*	*	*	*	*	*	*	*	*	*	*	*	*	*	*	*	*	*
5. Healthcare access	*	*	*		*	*	*		*	*		*	*	*	*		*	*	*	*	*	*			*	*

The presence of the star (*) indicates that the theme was mentioned or discussed in that particular interview.

## Data Availability

All data analysed in this study will be made available upon request from the corresponding author.
